# Round the Hospitals

**Published:** 1921-06-04

**Authors:** 


### ROUND THE HOSPITALS.

The visit of the King and Queen on Saturday last to the Whiteley Village, near Walton, has focussed attention on this remarkable social experiment, for so it may be called. Nurses are eligible for admission to the Village, and some are actually in residence. At a time when much thought is being bestowed on making provision in old age for members of the profession, the achievement of the Whiteley trustees is worthy of all praise. The gift of a million pounds to alleviate the trials of old age for some 300 persons gave an opportunity for realising theories founded on practical knowledge of the recluse life. The pettiness and isolation of the almshouses have been avoided by supplying lively communal interests. There is ample provision for care of the physical ills of age. Occupations and amusements are not wanting; independence, privacy and individuality have been safeguarded. The Whiteley Village touches in these particulars high-water mark among efforts previously attempted for clothing the declining years of men and women who have led busy lives with comfort and dignity.

The closing of seven wards at the London Hospital, which took place last week, involves certain rearrangements in the nursing staff, but no definite plans can be settled at the moment. Meanwhile, those sisters, nurses and probationers directly affected, some fifty upwards, have been readily absorbed into the hospital time-table. The nursing staff has hardly been kept up to its full strength lately, and the closing will enable the claims on the private nursing staff, on which the hospital has the first call, to be reduced.

Particulars of the Nightingale Fund scholarships are to hand this week. There are three open to any nurses trained in the Nightingale School who possess its certificate, and they are tenable for one year at King's College for Women, Campden Hill. The scholarships, which are of the value of £135 each, include board and residence at the College. An additional payment of £30 towards expenses will be made to each scholar, and this thoughtful provision will undoubtedly be much appreciated. Any nurse who desires to enter should send in her name to the Matron of St. Thomas's Hospital on or before June 30, 1921, giving her age, the date of her certificate, and a statement of the nature of the work on which she has been engaged since her training. The scholarships are intended to assist nurses to qualify for higher posts in their profession of which a larger number are offering for the nurse's choice each year.

The graves of sailors and soldiers and nursing sisters who died in the war were decorated with flowers according to American custom on May 30, "Decoration Day." It is a beautiful custom, and has taught us much in this country, where united commemoration of the dead had fallen a little into oblivion before the Great War. This year Commemoration Day was rendered notable in this country by a memorial service at St. Paul's, followed by the unveiling of a bust of George Washington, presented as "a token of the goodwill of the American people for the British people," in the chapel near to the tombs of Nelson and Wellington by the American Ambassador.

For some time the official activities associated with a Ladies' Association have been confined at Middlesex Hospital to a work guild and ambulance. The former, under the chairmanship of the Dowager Countess of Derby, has been very successful, and its last donation of garments totalled 1,200. On Thursday, May 27, at a meeting presided over by Princess Alice, Countess of Athlone, the wife of the Chairman of the hospital, it was resolved to form a Ladies' Association of much wider scope, comprising, in addition to the existing sections, separate ones for visiting the wards, entertainments, the library, and garden, under the chairmanship respectively of the Dowager Lady Brassey, Lady Bessborough, Hon. Margaret Wyndham, and Mrs. Felix Davis. So as to ensure co-ordination between each section these ladies, with Lady Derby and Miss Moore (respectively chairman of the work and ambulance sections), will form an executive committee of which Lady Brassey will be the chairman, the Hon. Ursula Lawley vice-chairman; Miss Smallwood will act as honorary treasurer to the Ladies'

**ROUND THE HOSPITALS—(*continued*).**

Association and Mrs. Webb Johnson honorary secretary. Membership of the Association is fixed at 10s. 6d. and associateship at 2s. 6d. Country as well as London members or associates will be welcomed, and it is hoped that the activities will extend to supplying country produce, flowers, fruit, eggs, etc., as well as the more active forms of help specified above.

The Liverpool Royal Infirmary was *en fête* on May 25, when the Ladies' Linen League held its annual meeting with a show of linen, and prizes were distributed to the nurses successful in their examinations. The membership of the League has risen to 428 and the articles contributed to over a thousand. The President of the League, Mrs. Corbett Lowe, was in the chair. She retires this year, and Lady Derby was elected in her stead, with Lady Stanley as Vice-President. Colonel Shute, Chairman of the Nursing Committee, spoke warmly of the nursing staff, of whom the governors are justly proud, and who, under the able guidance of Miss Cummins, are responsible in no small measure for the success of the hospital. He spoke with regret of the nurses' unsatisfactory quarters, and declared his determination that the first sod on the site of the new nurses' home should be cut this year. The building is estimated to cost about £110,000. The prize-giving was notable on account of the fact that three nurses passed out equally first in surgical nursing, necessitating extra prizes.

We hope the London Hospital Nurses' Bazaar, to be held in the Hospital Garden on July 6, at which Queen Alexandra has promised to be present, will reap the full benefit of the sympathy that has been evoked for the "London." The nurses have for some time devoted their off-duty, their talents, and a wealth of original ideas to the organisation of the Bazaar, in which they have been freely helped by the whole staff, from the house governor and matron down to the humblest laundry-maid. Thus a gift of 5s. to the head of the laundry, expended in materials for the manufacture of sweets, has produced £11, and throughout the institution the greatest ingenuity has been utilised to provide funds to finance the bazaar. It deserves to be a complete success, and we prophesy that the two stalls which have been set aside for articles made by the nurses will be the greatest success of all. Originality, skill, brains, and, the most valuable quality of all, sympathy have been employed in the fashioning of the things for sale, while an additional merit will be the inexpensiveness of the materials; even such unpromising articles as mustard barrels have been converted into most attractive waste-paper baskets.

The National Poor-law Officers' Association had the matter of bonuses brought very forcibly before them on the occasion of the annual meeting by Mr. J. J. Simpson, who urged that steps should be taken to secure from the Guardians something like stabilisation of salaries. Mr. Simpson believes that by negotiation with the Guardians permanent remuneration more adequate than before the war could be secured. The present scale of bonuses expires in September, when a general reduction is anticipated. The important thing is to begin already to talk about commuting the bonus and adding it to salary. This is a matter which affects a large number of our readers, and there is ground for satisfaction that Poor-law nurses have the College of Nursing to safeguard their interests.

As we go to press the Nine Days' Fair, from June 2 to 11, at the Bingley Hall, Birmingham, of which full particulars were given in our columns last week, is in full swing. On June 6 Princess Mary will receive purses from those who have been collecting on behalf of the Centre, and many presentations will be made to her. The visit of the Viscountess Cowdray the following day will also be a very popular event, and on both occasions a very pretty ceremony will take place, for the nurses belonging to the Birmingham Centre and the three surrounding counties are to assemble in uniform to greet the guests. The Hon. Sir Arthur Stanley, who takes a keen interest in the affairs of this Centre, has promised to attend on the 6th and 7th inst., and all omens are favourable for a most successful Fair.

The Northallerton Guardians have added ten years to the service of Nurse Elliott for superannuation purposes, whereby she retires "broken down entirely in the service of the Guardians," after twenty-four years of work, on the princely sum of £38 3s. 4d. a year. One of the Guardians present remarked that the remuneration of the nursing profession was at any time the worst in the country, and spoke cordially of Nurse Elliott's kindness and devotion to duty. The need for some more adequate scheme of superannuation was never more forcibly illustrated.

Armand Jeanne, the Belgian, accused of betraying Miss Edith Cavell to the German Governor, has been charged with the crime on the evidence provided by certain documents left by the Germans in a locked desk at Cambrai. When brought before the Juge d'Instruction, Armand Jeanne was identified by a former Red Cross nurse, Mlle. Nade, who testified that he came to Nurse Cavell as a Belgian soldier, was sheltered by her for three days and nights, and was then sent, with others, to the frontier. Afterwards he came a second time, and was again sheltered and helped. He was in German pay, and having learned Nurse Cavell's secret he betrayed her to the Germans and accompanied them to arrest her. Mlle. Nade replied to the prisoner's denials with the calm assurance "It is true; I myself conducted you to a retreat where you were hidden by Nurse Cavell's orders." Among the documents is said to be a lengthy report of Nurse Cavell's doings compiled by "Armand Jeanne of Mons." There are some fifty cases in which Belgian, French, and British subjects were sentenced on his information.

